# Stay-Green Trait: A Prospective Approach for Yield Potential, and Drought and Heat Stress Adaptation in Globally Important Cereals

**DOI:** 10.3390/ijms20235837

**Published:** 2019-11-20

**Authors:** Nasrein Mohamed Kamal, Yasir Serag Alnor Gorafi, Mostafa Abdelrahman, Eltayb Abdellatef, Hisashi Tsujimoto

**Affiliations:** 1 Arid Land Research Center, Tottori University, 1390 Hamasaka, Tottori 680-0001, Japan; yasirserag@tottori-u.ac.jp (Y.S.A.G.); meettoo2000@tottori-u.ac.jp (M.A.); 2 Agricultural Research Corporation, Wad-Medani P.O. Box 126, Sudan; 3 Botany Department, Faculty of Science, Aswan University, Aswan 81528, Egypt; 4 Commission for Biotechnology and Genetic Engineering, National Center for Research, Khartoum P.O. Box 6096, Sudan; eltaybfarah@gmail.com

**Keywords:** stay-green, drought stress, heat stress, quantitative trait loci, yield

## Abstract

The yield losses in cereal crops because of abiotic stress and the expected huge losses from climate change indicate our urgent need for useful traits to achieve food security. The stay-green (SG) is a secondary trait that enables crop plants to maintain their green leaves and photosynthesis capacity for a longer time after anthesis, especially under drought and heat stress conditions. Thus, SG plants have longer grain-filling period and subsequently higher yield than non-SG. SG trait was recognized as a superior characteristic for commercially bred cereal selection to overcome the current yield stagnation in alliance with yield adaptability and stability. Breeding for functional SG has contributed in improving crop yields, particularly when it is combined with other useful traits. Thus, elucidating the molecular and physiological mechanisms associated with SG trait is maybe the key to defeating the stagnation in productivity associated with adaptation to environmental stress. This review discusses the recent advances in SG as a crucial trait for genetic improvement of the five major cereal crops, sorghum, wheat, rice, maize, and barley with particular emphasis on the physiological consequences of SG trait. Finally, we provided perspectives on future directions for SG research that addresses present and future global challenges.

## 1. Introduction

**Figure 1:**
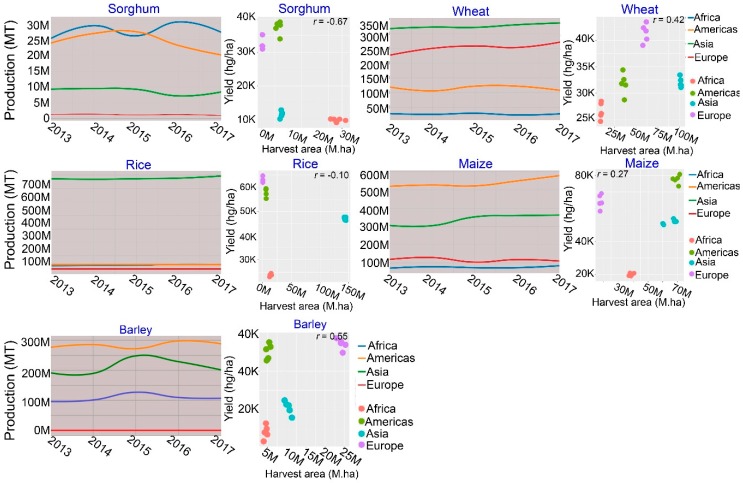
Total production (million tones, MT) and yield (hectogram/hectare, hg/ha) of major cereal crops, including sorghum, wheat, rice, maize and barley. Total production and scatter plot of the relationships between yield (kg/ha) and harvest area (ha) in the five major cereal crops. Correlation coefficient calculated by the Pearson method. The data was obtained from FAOSTAT (http://www.fao.org/faostat/en/#data/QC) database, accessed September 2019 [1].

**Figure 2:**
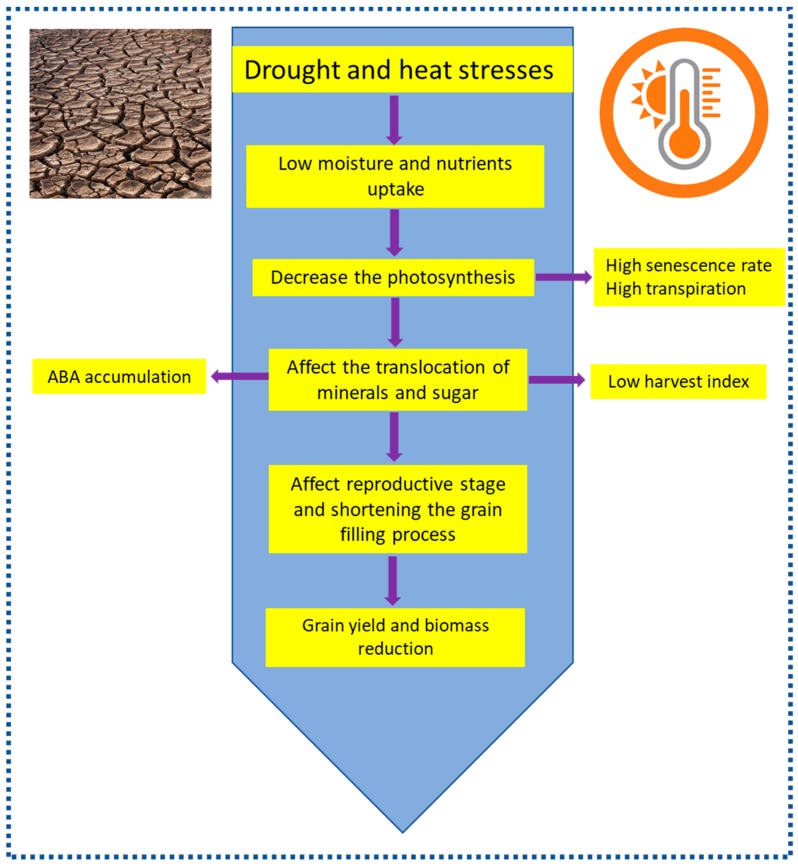
The plant features under heat/drought stress relevant to crop biomass and yield [4,5,6].

Sorghum (*Sorghum bicolor* L. Moench), wheat (*Triticum aestivum*), rice (*Oryza sativa*), maize (*Zea mays*), and barley (*Hordeum vulgare* L.) are considered as major staple foods for a large portion of the world population [1] (Figure 1). However, global food security is being haunted by the rapid increase in the world population and drastic changes in the climate [2,3,4]. For instance, heat and drought are the two most important environmental stresses imposing huge impact on crop growth, development, grain yield, and biomass productivity [4,5,6] (Figure 2). With the increasing expectations of crop yield losses because of the global climate change and the exponential population growth, there is an urgent need to accelerate plant breeding and mining of novel traits for increased yield potential and better adaptation to abiotic stresses to secure the food availability and meet the future demand for agricultural production [7]. In this context, stay-green (SG) genotype selection can be a principle strategy for increasing crop production to meet the mandate of an expected increase in population, particularly under heat and water-limited conditions.

SG genotypes constitute a potential germplasm source for the genetic improvement of important crops to mitigate heat and drought stresses. SG genotype is characterized by delayed senescence because of chlorophyll (Chl) loss compared with a non-SG standard genotype. Therefore, SG is considered as an important agronomic trait that allows plants to maintain their leaves photosynthetically active and subsequently improved the grain-filling process even under stress conditions [8,9,10,11,12,13,14]. SG has two types, functional and non-functional. The functional SG genotypes are agronomically important as they are able to maintain their photosynthetic capacity compared with the non-SG genotypes. The functional SG genotypes delay the onset of senescence (type-A) or initiate the senescence on schedule, but proceeds more slowly (type-B) [15]. In the non-functional/cosmetic SG genotypes the senescence is initiated on a normal time-scale, however, leaf greenness is maintained because of the failure of the Chl degradation pathway with decline in photosynthetic capacity (type-C), or leaf pigment remain because of freezing or drying such as frozen spinach or herbarium specimen (type-D), or the intensely green genotype may have normal ontogenetic photosynthetic capacity but their absolute pigment contents classified it as a SG type-E [15,16,17,18,19]. SG has been used initially as a phenotype descriptor by legume breeders in *Vicia faba* [20], and later on, it was established as a superior characteristic and marketing feature for many commercial grain crops [11]. 

**Figure 3:**
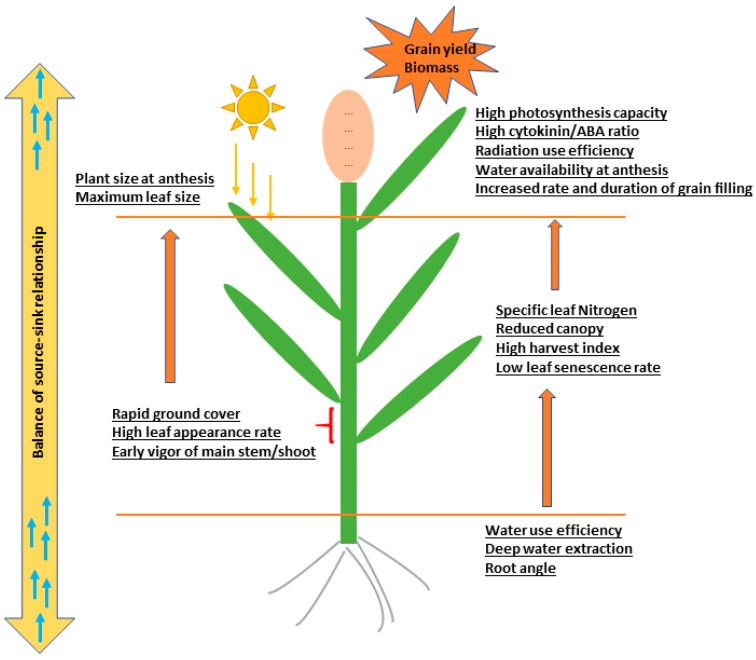
The physiological features of the stay-green plants including photosynthesis, transport of photosynthates and source-sink relationship. These physiological parameters operate to determine grain yield and biomass [11,12,13,24,29,40,56].

In addition to the beneficial roles of SG trait in yield improvement and tolerance against drought and heat stresses [11,12,13,21,22,23,24,25], desirable morphological traits associated with SG trait, including a greater number of grains per ear [22], enhanced resistance to stem lodging [26], and greater tolerance to biotic stress such as spot blotch infection [27,28] have been reported. An increase in leaf area, rate and duration of grain filling and photosynthetic competence, water use efficiency, leaf anatomy, have been found to be a characteristic for the SG trait [24,29] (Figure 3). It has been reported that breeding with a SG phenotype can improve yield under post-flowering drought stress (terminal drought stress), without yield penalties in environments not affected by drought [24,30,31].

**Figure 4:**
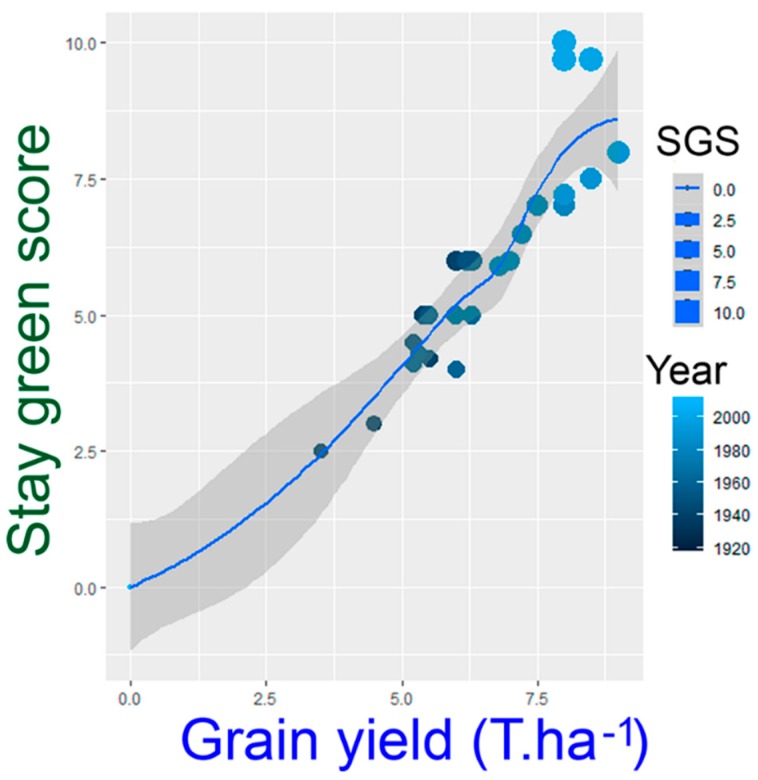
The relationship between the increases in yields and stay-green scores of maize varieties produced since 1930 according to [15], illustrating the contribution of the SG in increasing the yield.

Quantitative trait loci (QTL) mapping for SG has been performed for the major five cereal crops, wheat [32,33], maize [34,35], rice [36,37], sorghum [38,39], and barley [22]. In maize, the utilization of SG trait in breeding programs results in significant genetic progress for high grain yield and tolerance to abiotic stress (Figure 4). Understanding the physiology underlying the SG trait will facilitate the identification of functional markers and/or genes for adaptation to limited water and heat stress environments. Therefore, there is a need to increase knowledge of the SG potentiality to increase grain yield under drought and heat stresses in cereals and to explore this trait extensively in breeding programs to harness more advantages of this trait. In this review, we summarize and discuss the recent progress in the application of SG trait as a breeding target under high temperature and water-limited conditions in sorghum, wheat, rice, maize, and barley. This review has aimed to shed light on the main aspects of the SG applications for plant breeding in cereals, with assertion on the physiological consequences of staying green, and its potential use to improve yield under drought and heat stress environments.

## 2. Stay-Green in Sorghum

### 2.1. Stay-Green QTLs in Sorghum

Drought stress is often a limiting factor for sorghum production and can lead to complete crop failure [40]. In the long history of sorghum breeding for drought adaptation, SG is the best-characterized trait contributing to drought adaptation in sorghum [12,13,21,38,41,42,43]. Different genotype sources for SG trait have been identified in sorghum, including “B35,” “SC56,” and “E36-1” with “B35” genotype being the most popular [13,44,45,46].

**Figure 5:**
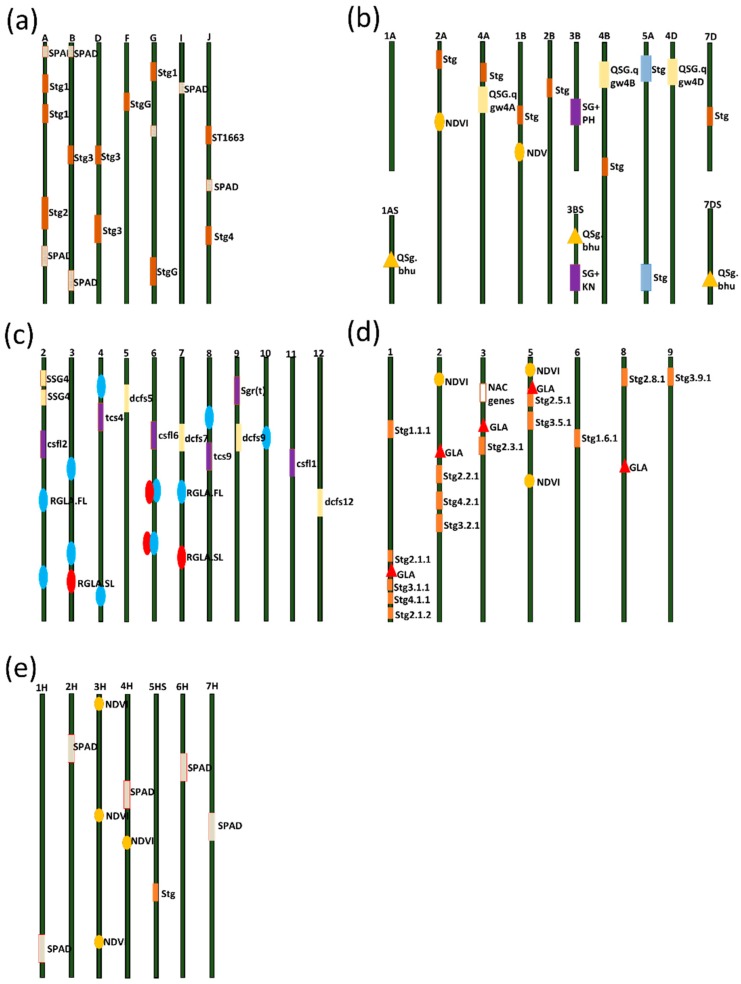
This schematic diagram illustrates the major stay-green quantitative trait loci (QTLs) mapped (based on the previous literature) in the five major cereal crops; sorghum (**a**), wheat (**b**), rice (**c**) maize (**d**), and barley (**e**). In sorghum, the same QTLs are mapped in different populations. In wheat, different stay-green QTLs indicated with different colors are mapped in different populations. Stg denotes stay-green, PH denotes plant height, and KN denotes kernel number. In rice, different colors denote QTLs identified by different groups, RGLA.FL denotes retention green leaf area of flag leaf; RGLA.SL denotes retention green leaf area of the second upper leaf; SSG4 denotes stay-green; dcfs denotes degree of chlorophyll content in flag and second leaves; tcs denotes total cumulative SPAD value of the four upper leaves; csfl denotes cumulative chlorophyll content of flag leaf. In maize, GLA denotes green leaf area, NDVI denotes normalized difference vegetation index.

QTLs for SG have been identified in the three source lines using several bi-parental populations. Four QTLs for the SG trait has been identified, following analysis on a recombinant inbred line (RIL) population produced from the cross between “B35” (SG line) and “Tx7000” (senescent, post-flowering drought-sensitive) [44,47]. Among these QTLs, *Stg*1 and *Stg*2 QTLs have been mapped to chromosome 3, whereas *Stg*3 and *Stg*4 QTLs have been located on chromosomes 2 and 5, respectively (Figure 5a) [44,45,47].

The four SG QTLs combined explained 53.5% of the phenotypic variation within the “B35” × “Tx7000” RIL population [45]. Following these efforts, several QTLs contributing to SG phenotype expression under drought have been validated across different research groups (Figure 5a) [21,38,39,44,45,46,47,48,49,50]. However, the four QTLs identified in “B35” are the most stable and significant, and are currently being introgressed in several genetic backgrounds through marker-assisted breeding (MAS) [21,51,52]. Several studies reported a close association between the SG phenotype and plant response to stress, as illustrated in the co-localization of QTLs for SG with QTLs for temperatures and drought stress tolerance [15,44,53,54]. Another example of QTL co-localization was found in studies on RIL populations derived from an original cross between lines with different nodal root angles (narrow vs. wide-angle) [55]. These nodal root angle QTLs have been found to overlap with SG QTLs. Co-localization between SG and root suggested that modified root architecture is likely to be a contributor to the SG trait observed in this population, which will enhance the water extraction capabilities, especially under stress conditions [11,53]. Therefore, selection for SG QTLs can simultaneously lead to the inheritance of stress tolerance features [15,53].

### 2.2. The Physiology of Stay-Green in Sorghum

Considerable efforts have been made to understand the physiological mechanism of the SG in sorghum. The earlier studies demonstrated the role of the N uptake by the SG and non-SG sorghum hybrids [41]. SG hybrids grown under terminal drought stress were able to balance between the N demand by the grain and N supply during grain filling. In these hybrids, at anthesis, the leaf N content was correlated with the onset and rate of leaf senescence under terminal drought stress [41]. On the other hand, it is reported that SG loci influences the root architecture and increases the water accessibility during grain filling under water-limited field conditions [57].

Recently it has been reported that adaptation of SG sorghum to drought is a consequence of canopy development, leaf anatomy, root growth, and water uptake and utilization [11,12,13,53,58]. For example, [21,53] showed that SG QTLs impact water uptake, transpiration efficiency, and grain yield; however, this impact depends on the genetic background and the environment (Figure 2). Furthermore, at flowering stage SG QTLs modify tillering, leaf number, and leaf size, and thus reduce the canopy size. With this small canopy size at flowering, SG reduces pre-anthesis water use (Figure 2 and Figure 3) and increases water availability during grain filling, which in turn increases grain yield under post-flowering water stress [11,12]. Grain yield can be increased with just small increases in water use during grain filling. From simulation studies in sorghum, it is reported that addition of 1 mm transpired water during grain filling could increase the grain yield by about 30 kg ha^−1^ [59]. A recent report by [13] suggested that SG QTLs introgressed from “B35” into Sudanese background “Tabat” can regulate their transpiration rate and water utilization depends on the drought severity. All this information indicates that SG trait increased water availability after anthesis and caused a delay in leaf senescence, which subsequently improved the yield in sorghum. Thus, it is possible to consider that SG phenotype is a result of the interaction between SG loci that largely regulate the plant size, and hence water demand and utilization by the crop, and the environment that regulates water supply by the soil. Based on all these research efforts, SG could be considered as a harmonized system that operates to make available the necessary water for growth and production under terminal drought stress. On the other hand, because SG phenotypes increase water availability, the photosynthesis rate, harvest index, and biomass of the SG introgression lines were better than non-SG phenotypes [13]. These recent investigations illustrated the high productivity of the SG genotypes under the terminal drought stress in sorghum. Interestingly, the positive impact of SG on yield across multiple genetic backgrounds was reported by [60]; and all four SG QTLs increased the grain yield under drought as well as under well-watered conditions without yield penalty under well-watered conditions [13,41,61]. In sorghum, only enhanced water productivity (TE) could simultaneously improve grain and stover yield along with the crop resilience [40] (Figure 3).

Recently, metabolic and transcriptomics studies are able to provide further insights into the molecular and physiological basis of the SG trait by identifying differential expression of specific genes across different varieties or in response to a change in the environment [62,63,64]. These techniques can reveal more about the processes involved in the SG phenotype, without needing to identify the genes underlying the QTLs. Few studies have used transcriptomic approaches to analyze the change in gene expression in sorghum as a result of abiotic stress such as osmotic stress and abscisic acid treatments [62,65,66]. Johnson [64] compared the transcriptome of “B35” (SG) and “R16” (senescent) plants grown in non-stress conditions. In the “B35” line, 1038 genes were upregulated and 998 genes were downregulated compared with “R16.” However, there is no study that utilized the near-isogenic lines of the four SG QTLs and illustrated the differences between the plants under the stress and non-stress conditions. This area of research remains untapped; however, it can provide more detailed information about SG, improve our understanding, and enable more efficient opportunities to deploy SG in breeding and crop improvement. It is anticipated that the genes regulate the SG in sorghum can be modulated in the other major cereals (wheat, maize, and rice) to improve their adaptation to drought wherever water is limited after flowering.

Although sorghum is predominantly grown in the arid and semiarid regions of the world, where heat stress is known to induce significant yield losses, very few reports are handling the heat stress and combined drought-heat stress issues [63]. Recently Tacka [64], from 29 years field-trial data spanning 408 hybrid cultivars, suggested warming scenarios break down, and identified a 33 °C as a temperature threshold, after which, yields start to decline. They suggested that both pre- and post-flowering stages were equally important for overall yields; furthermore, they concluded that the introduction of wider genetic diversity for heat adaptation into the ongoing breeding programs will facilitate sorghum resilience under climate change [64]. As SG trait improved grain yield in sorghum under terminal drought stress, it can also tolerate high temperatures better than non-SG crops, but no report described the SG as adaptation trait for heat and combined heat-drought stresses in sorghum, which remains a future task.

## 3. Stay-Green in Wheat

### 3.1. Stay-Green QTLs in Wheat

Compared to sorghum, in wheat, the progress in SG research is relatively small. On the other hand, compared to drought in sorghum, in wheat, the SG has been studied intensively as one of the adaptation traits for heat stress, the major abiotic stress affecting yield in wheat. Kumar [32] identified three QTLs on chromosomes 7DS, 3BS, and 1AS using a recombinant inbred lines (RIL) population between the SG “Chirya 3” and non-SG “Sonalika” under natural field conditions (Figure 5b). He suggested that cultivars with SG characteristics offer a better option for high-temperature and drought environments, and the identified QTLs provide initial information to generate a finer map and to recruit a marker-assisted selection (MAS) strategy. A high-density genetic map consisting of 2575 markers constructed by Shenkui [67] was used to map QTLs of SG and other agronomic traits under four different water regimes. A total of 108 additive QTLs were identified. Twenty-eight QTLs were for Chl content detected on 11 chromosomes, 43 were detected for normalized difference vegetation index (NDVI) on all chromosomes except 5B, 5D, and 7D [67]. 

Interestingly in many cases, the SG QTLs in wheat found to be co-located with QTLs for other important traits, the thing that can allow the simultaneous selection and improvement. For example, Huang [68] detected SG QTL on chromosome 3B in a similar linkage group where a QTL for plant height is positioned. Also, the SG QTL and a QTL for kernel number per spike were identified in the same region on chromosome 3BS [69] (Figure 5b). Pinto [70] identified a total of 44 loci linked to SG and related traits, spread through the genome. Of these 44 loci, those on chromosomes 1B, 2A, 2B, 4A, 4B, and 7D possessed the strongest and most repeatable effects. Pinto [70] showed that the association of the SG trait and all SG-related traits with stress tolerance is reinforced by results demonstrating that the same genomic regions have an effect on kernel number, yield, grain weight, NDVI, canopy temperature, and also the rate and length of grain-filling. Christopher [24], identified SG QTLs associated with QTLs for seedling root number and *Rht*-height genes. These findings of the co-location of QTL for SG and performance traits confirms the usefulness of SG for productivity enhancement under heat and drought stresses, and suggest avenues for further research to clarify the physiological and genetic mechanisms of SG for better understanding and exploiting SG in wheat. 

The moderate and greater effect of each SG QTL is reported in wheat and other cereal crops. Three QTLs identified for SG in wheat explained up to 38.7% of phenotypic variation in a study by [32]. In maize, single SG QTL explained from 3.2 to 12.5% of the phenotypic variance [71]. In sorghum, on the other hand, each of the four key SG QTLs possessed a considerably higher percentage of variation, 10 to 30% [47]. Accordingly, in comparison with other crops, it is possible to speculate that SG in wheat is a function of several genes with relatively small effects. The identification of genetic loci regulating SG in a wheat mapping population offers the tools to enable MAS to accelerate and advance the competence of plant breeding. However, to what extent these markers can be largely applicable is still under examination. As the genetic mechanisms controlling SG and yield-associated traits are very complex, the use of high-density linkage map will enable exploration of novel favorable alleles. In wheat only and not the other species physical deletion maps are useful for physically allocating ESTs and genes to small chromosomal regions for targeted mapping. Sourdille [72] improved the usefulness of deletion stocks for chromosome bin mapping and characterized 84 deletion lines covering the 21 chromosomes of wheat by 725 microsatellites. Several genes and QTLs have been physically mapped on the deletion maps. These deletion stocks could potentially help to elucidate the location and if possible, the cloning of SG genes and other associated traits.

### 3.2. The Physiology of Stay-Green in Wheat

A positive correlation between yield and SG phenotypes has been recognized under heat and drought stresses, and non-stress conditions [70,73,74,75,76]; however, negative effects on yield have rarely been reported [77,78]. Pinto [70] showed that SG trait was positively associated with yield and yield components in bread wheat grown under both heat-stress and non-heat stress conditions. Christopher [73] showed that SG genotypes exhibited higher mean grain mass and showed small variances in water use before anthesis, or better water extraction from depth after anthesis. The deep soil moisture was depleted, indicating that the extraction of deep soil moisture was essential for adaptation of the SG genotypes. However, it is clear from their study that mechanisms other than root traits also exist. 

The usage of SG trait alone or in combination with other markers/traits related to water stress has great potential for selecting either for specific or broad water-stress adaptation [24,79]. Christopher [24] reported that SG traits integrating senescence, plus time from anthesis to onset, mid-point, and near completion of senescence were positively correlated with high yield in the severe and mild water-stress environment. He finally suggested that these traits have prospective to surge the rate of progress toward higher yield with better yield stability of wheat in a wide range of environments. The improvement of molecular markers for the selection of these traits would be highly needed and will enable selection in early generations. Although many traits such as canopy temperature depression (CTD) have been suggested in early studies as selection criteria to assess heat tolerance [80,81], published studies on a possible association between the SG trait and CTD in the different crops are scarce.

In durum wheat, a SG mutant has been characterized with increased rate and duration of grain filling, leaf area, and photosynthetic competence [29]. During the grain development, flag leaf SG duration and harvest index showed positive relationships with water use efficiency [82]. Under field conditions, senescence has been quantified by NDVI and linked with yield and response to drought and heat [70,74,83]. Several studies [84,85] demonstrated that a decrease in late-season leaf senescence in wheat accessions was correlated with increased yield. The late-season maintenance of Chl and reduced senescence, slow down the decrease in photosynthetic capacity (i.e., RuBP regeneration and Rubisco activity,) and resulted in a longer photosynthesis duration and higher production potential [29,86]. In a study by [87], delayed flag leaf senescence was associated with late heading date and high grain yield in three water regimes. Bogard [75] showed a negative correlation between anthesis date and the onset of senescence and between leaf senescence and grain yield, which explained by associations between QTLs affecting leaf senescence and QTLs for anthesis date [70,75]. Liang et al. [87] provided evidence that grain yield was sink-limited in three different moisture levels until the final stages of growth, at that time positive relationship between grain yield and light-saturated net carbon assimilation at anthesis and negative relationship between grain yield and flag leaf senescence indicated that sustained photosynthesis contributed to additional grain filling that increased grain yield. Their results implied that delayed leaf senescence and late-season photosynthesis were driven by the size of the reproductive carbon sink, which is greatly controlled by factors affecting the grain numbers.

Under heat and combined heat-drought stresses, SG calculated based on NDVI at physiological maturity and the rate of senescence showed positive and negative relationships with yield, respectively. In addition, canopy temperature at the mid-grain-filling stage and SG variables accounted for ~30% of yield variability in multiple regression analysis, suggesting that SG traits may offer cumulative effects, together with other traits, to improve adaptation under heat stress [88]. 

Few studies dealt with large-scale phenotyping of SG and early senescence phenotypes under field conditions. Sebastian [89] estimated the onset of senescence of flag leaves in 50 winter wheat cultivars using spectral remote sensing tool as a high-throughput phenotyping tool, and identified the SG and the early senescence phenotypes. Recently, [90] using a handheld color spectrometer successfully converted spectra of the whole canopy into color values measured at the flag leaf level. They confirmed that spectral remote sensing is a suitable method for the high-throughput phenotyping of flag leaf senescence. The work of [24] showed how reliably the SG trait value could be predicted for use in breeding through phenotyping together with environmental simulation and characterization. On the other hand, the selection of functionally SG germplasm from large breeding populations can be achieved easily by complementing the use of NDVI with the new high-throughput phenotyping tools that have the ability to precisely monitor changes in leaf area, greenness and photosynthetic activity (via changes in canopy temperature) [91].

The SG characters are genetically complex with environmental influences that require further exploration, therefore, in the wheat understanding of SG genetic control, and the QTLs expression in different sets of environments would ease the selection for the trait. By measuring late-season photosynthesis or simply select genotypes with higher grain numbers, which is likely to be associated with the ability of the plant to remain photosynthetically active late in the growing season under optimal and stress conditions, wheat breeders might be able to selected genotypes with improved grain yield. The high-throughput phenotyping methods will facilitate the uncovering of senescence mechanisms of cereal plants in huge field trials and helps to better identify the impact of the senescence on grain yield and grain protein content.

## 4. Stay-Green in Rice

### 4.1. QTLs for the Stay-Green Trait in Rice

Four QTLs in rice (*Oryza sativa*) (*Csfl12, TCS4, Csfl6, and Csfl9/Tcs9*) were detected in two RILs populations obtained from the combination of “Suweon490” (japonica and synchronized) x “*SNU-SG*1” (japonica and SG) and “Andabyeo” (India and synchronized) x “*SNU-SG1*” (Figure 5c). Identification of the SG QTLs Csfl6 and Tcs9 in the same positions with the two-grain yield QTLs (Yld6 and Yld9) strengthens the connection between the presence of SG and high productivity in rice [92]. For Chl content, [93] reported six QTLs on five chromosomes using backcross lines, and [94] reported other three QTLs on three chromosomes using a doubled haploid (DH) population obtained from “japonica × indica” hybrid. Jiang [95] analyzed the genetic basis of SG using DH lines obtained from “indica × japonica” hybrid and detected 46 main-effect QTLs in 25 chromosomal regions and 50 digenic interactions concerning 66 loci on 12 chromosomes. Yue [36] identified more than 30 QTLs for flag leaf traits, degree of greenness and SG-related traits, of which 10 QTLs were consistently detected in different years. They reported that region RM255-RM349 on chromosome 4 controlled the three-leaf morphological traits (leaf length, width and area) simultaneously and explained a great part of the variation, which was useful for the genetic improvement of grain yield. The region RM422-RM565 on chromosome 3 was linked with SG traits, although the utilization of this region in breeding needs to be evaluated by constructing near-isogenic lines [36]. Lim [96] identified the main-effect of QTLs for the functional SG traits in the japonica rice SNU-SG1 and isolated candidate genes. They carried out QTL analysis using 131 molecular markers with F_7_ RILs from a cross of japonica rice “*SNU-SG1*” and indica rice “Milyang23 (M23).” They identified 18 QTLs for eight traits related to the physiological response of the SG which provide valuable data for breeding high yielding rice.

A recessive *sgr* mutant has been isolated and mapped on chromosome 9. It delays the process of senescence but does not maintain photosynthetic capability [97]. After this, several natural variants or mutants exhibit SG in rice has been reported, such as nyc1 [98], nyc3 [99], SGR [100], and nol [16]. Rice SG mutant retains Chl b and LHCII (light-harvesting Chl-binding protein complexes of PSII) in the light as well as in the darkness [101]. However, Chl *a* as well as other Chl–protein complexes decrease during senescence in this mutant. Jiang [100] isolated another 60Co γ-rays induced rice SG mutant. The gene of this mutant was cloned by a positional cloning strategy and is found to be an allele of the SG rice gene sgr reported by [97].

About 132 rice senescence-associated genes (*SAG*s) allocated on all the 12 chromosomes have been annotated in the leaf senescence database (http://psd.cbi.pku.edu.cn/). These *SAG* genes are classified into five groups: (a) natural, (b) dark-induced senescence, (c) nutrition deficiency-induced senescence, (d) stress-induced senescence, and (f) others [102]. The corresponding mutants of SAGs can be divided into two key categories according to their phenotypes: delayed senescence mutants and premature senescence mutants [103]. However, there are much more premature senescence mutants have been reported in rice compared with the delayed senescence mutants, for instance, the noe1 [104], ospse1 [103], psd128 [105], es1-1 [106], lts [107], rls1 [108], and ps1-D [109] mutants, are involved in different complex regulatory networks of senescence. [100] proposed that SG rice mutant *sgr* involved in regulating or taking part in the activity of pheophorbide an oxygenase (PaO), and then may influence Chl breakdown and degradation of pigment–protein complex. From ethyl methane sulfonate (EMS) mutant bank of rice cultivar Zhong Jian100, an additional five premature leaf senescence mutants (psl15, psl117, psl50, psl89, and psl270) were identified [37]. The influence of these mutations on the agronomic traits as well as the physio-biochemical properties including chloroplast structure, Chl contents, photosynthetic ability, expression profile of ABA, and senescence-related genes, response to darkness and ABA, and the genetic controls of their premature senescence phenotypes were investigated [37]. These results obtained by He [37] provided the basis for the isolation of these premature senescence genes and the elucidation of the senescence mechanism in rice. Zhao [19] from genome wide association analysis (GWAS) of 368 rice accessions reported 25 known genes, among which the pleiotropic candidate gene *OsSG1* accounted for natural variation in Chl content and SG. Further analysis indicated that the significant phenotypic differences between alleles are caused by 20 large-effect, non-synonymous SNPs within six known genes around GWAS signals and three SNPs in the promoter of OsSG1 [19]. Moreover, [19] found all OsFRDL1 and CHR729 haplotypes in wild rice, and OsFRDL1-1 and CHR729-2 haplotypes were prevalent in japonica rice, whereas OsFRDL1-3, OsFRDL1-2, and CHR729-1 haplotypes predominated in Indica rice. They concluded that during domestication of japonica the cultivated areas progressively extended from low to high altitudes along with the variations in light intensity and day length. During this adaptation, new natural mutations for higher SG and Chl were maintained and gradually accrued along with natural elite variation from wild rice [19]. Interestingly, the 368 rice accessions showed no significant correlation between Chl content and SG. The Chl content was higher and SG was stronger in japonica than in Indica [19]. 

### 4.2. The Physiology of Stay-Green in Rice

The SG trait in rice cultivars is known as the ability to maintain green leaves and benefits dry matter production in drought-prone areas [110]. Chl degradation and the disassembly of the photosynthetic apparatus were the most remarkable phenomena in leaf senescence, which result in decreases in photosynthetic capacity, energy, and efficiency. Also, there is a considerable decline in electron transport chain for the remaining components in the leaf [111,112,113]. From their research on rice, [114] reported that the major advances in understanding the origins of SG occurred after the elucidation of the Chl catabolism pathway and the associated genes, which pointed out the functional significance of the photosynthetic and N remobilization phases of leaf development. According to Kusaba [115], continuous biosynthesis of Chl in excess of the activity of the catabolic pathway provide another way to SG. In rice, several studies have been conducted at the molecular level to clarify the leaf senescence process. Several genes involved in leaf senescence have been identified, including hormonal factors, transcription factors, phytochrome B, the Chl degradation genes, and defense-related proteins. Moreover, a comprehensive understanding of leaf senescence was achieved by a time-course gene expression profiling of leaves during the grain-filling period. Rong [116] reported that the overexpression of SG rice like gene (*SGRL*) reduces the level of Chl and Chl-binding protein in leaves, and accelerate their degradation in dark-induced senescence in rice leaves; therefore, they suggested that the *SGRL* protein is involved in Chl degradation. Moreover, the presence of conserved amino acid domain in SGRL and SG rice implies similar biochemical functions [114]. Mao [117] showed that overexpression of *OsNAC2* results in premature senility rice, which indicated the *OsNAC2* role in ABA-induced leaf senescence pathway. The results obtained by [37] have provided principles for further studies on the fine mapping, functional analysis, and isolation of the premature leaf senescence corresponding genes. The senescence-associated genes (*SAGs*), including the six *NACTFs* were identified from the gene expression profiling of the flag leaves from vegetative to senescence stages [37]. On the other hand, mapping of these *SAGs* into cellular processes enabled the identification of the key cellular mechanisms of the shared and differential senescence programs between flag leaf and second leaf. When the changes after panicle removal observed among the differential senescence programs, invariable core senescence programs were distinguished from the variable senescence programs. 

Although the contribution of the SG genotype to stable yield production under drought stress has been studied in other crops like sorghum [55], a robust relationship between grain yield increase and leaf greenness has not been reported yet in rice. In contrast, a negative correlation was reported [36,95]. The intra subspecies cross, japonica/japonica has been used by Fu [56] to investigate the inheritance mode and the genetic relationship between the SG traits and the yield and its components. They found that the correlation between the seed-setting rate and SG was higher than that between SG and yield, indicating that SG enhances the yield through the direct improvement of seed setting. In this case, the simultaneous increase of the source (photosynthetic rate) and sink (partitioning to grain) strengths is most likely to be the drive to achieve grain yield [56] (Figure 3). Park and Lee [118] measured Chl content and photosynthesis under light saturation (Pmax) in SNU-SG1 and other two rice varieties. They found that SNU-SG1 maintains high Chl content and photosynthetic capability for longer during the monocarpic senescence, and has improved seed setting rate. Thus, they concluded that SNU-SG1 could be used as a desirable genetic source of functional SG, in breeding programs to increase crop productivity.

## 5. Stay-Green in Maize

### 5.1. QTLs for the Stay-Green Trait in Maize

The genetic analysis of complex traits in maize under abiotic stresses has focused mainly on drought tolerance [119,120,121]. Only a few studies have been conducted to map the SG QTLs in maize. In temperate maize germplasm, [122] mapped three and five QTLs in F_4_ progenies and their test crosses, respectively. Zheng [34] mapped 14 QTLs in F_2:3_ progenies, and [70] mapped 14 QTLs in F_2_ plants. Zheng [34], reported that the respective QTL contribution to phenotypic variance ranged from 5.40% to 11.49%, with trait synergistic action from Q319. In tropical germplasm, Câmara [123] mapped 20 and 33 QTLs using F_2:3_ progenies from two populations. Additional QTL analyses indicated that multiple intervals of SG QTLs overlapped with yield QTLs. Yang [124] performed a QTL mapping by using 165 F_3:4_ recombinant inbred lines population derived from a cross between a SG inbred line (Zheng58) and a non-SG inbred line (B73) genotyped using 211 polymorphic simple sequence repeat markers. A total of 23 QTLs for Chl content, photosystem II photochemical efficiency, and SG area at maturity stage were mapped on nine chromosomes. The single QTL explained 3.7–13.5% of the phenotypic variance. They validated some important SG QTLs, which were significantly correlated with the plant yield. These studies provide a better insight into the mechanism that regulates leaf SG in maize and contributes to the development of novel elite maize varieties with delayed leaf senescence through MAS. Recently, Zhang [14] in a mapping population derived from the Illinois High Protein 1 (IHP1) and Illinois Low Protein 1 (ILP1) lines, identified a novel QTL controlling functional SG, showing different rates of leaf senescence. They further described the role of NAC7 (transcription factors) in improving functional SG and yield through the regulation of the resource allocation from vegetative source to reproductive organs. These findings of [14] highlight and draw attention to NAC7 as a core target for improving functional SG and yields in maize and other crops. Sekhon [125] studied a SG line able to maintain high grain filling and photosynthetic capacity for six additional critical days compared to a naturally senescent genotype and revealed the genetic architecture of senescence in maize. Furthermore, they placed nine candidate genes represent diverse processes, including sugar-mediated signaling (trps13), transport (*ZmSWEET1b* and *ZmMST4*), and control of sugar uptake (*dek10*) in one model, and elucidated the role of sugar partitioning and sugar signaling in inducing senescence [125]. 

QTL pyramiding in elite inbreeds, would have enhanced levels of SG in maize. MAS has been successfully used in maize for grain yield, as well as in other crops for other traits [126,127,128]. For functional marker development and rapid identification of candidate genes or loci, [129] suggested a strategy of combining Meta QTL (MQTLs) analysis and regional association mapping. Several maize orthologs of rice yield-related genes were identified in these MQTL regions [129]. Based on the results of the meta-analysis and regional association mapping, three potential candidate genes *GRMZM2G359974, GRMZM2G301884,* and *GRMZM2G083894*) associated with kernel size and weight within three MQTL regions were identified.

### 5.2. The Physiology of Stay-Green in Maize

Swanckaert [130] characterized two SG types in maize: SG type and normal type. The SG varieties characterized by higher photosynthetic capacity values coincided with higher values for the proxies. Although a higher photosynthetic capacity did not induce the higher accumulation of assimilates in the leaves, the SG trait was characterized as a cosmetic SG. The SG trait influenced N dynamics in the plant since the lower translocation of N from the leaves to the ear resulted in low N concentration in the ear and consequently lower ear dry matter yield. No differences found either in whole-plant N concentration or whole-plant dry matter yield. As the SG trait mainly cause shifts in dry matter partitioning and N balance between vegetative and reproductive tissues, the energy source also shifts from starch (from ear source) to cell wall material (from stover source) [130].

SG maize cultivars have been selected to maintain a high green leaf area during the post silking development phase. Consequently, they are able to sustain higher photosynthetic capacity than non-SG cultivars at a time of high demand for photosynthates [131,132]. This stage is critical because the highest amount of dry matter partitioned to the grain is accumulated after silking [133]. A SG line P3845 showed a delay in leaf senescence correlated with increased levels of Chl, when compared to a non-SG line Hokkou 55, [134]. P3845 showed high levels of cytokinins (trans-zeatin riboside, t-ZR; dihydro zeatin riboside, DHZR; isopentenyl adenosine, iPA) and low level of ABA in the leaves. On the other hand, in roots, P3845 showed increased levels of t-ZR, DHZR, and ABA, but decreased concentrations of iPA [134]. Therefore, [134] concluded that the delayed senescence in P3845 is a result of the higher rate of cytokinin transport from roots to leaves, and the translocation of ABA from roots to shoots may be plugged in the SG cultivar, which results in leaf senescence retardation [134].

In maize, the SG trait used to be evaluated at the leaf level using portable Chl meters, such as the Minolta SPAD. Chl content and imaging spectroscopy were also used to evaluate SG trait in maize [135,136,137]. The measurement of the NDVI during canopy development stages was proposed as a secondary trait to be included in maize breeding to indicate early vigor and grain yield under drought and well-water conditions [138,139]. The SG trait should be considered in maize breeding programs; it accounts for grain yield and other important related traits of agronomic/economic importance, particularly drought tolerance. However, information on the inheritance of the SG trait in maize is limited. Many reports have shown that maize breeding programs aiming at improving grain yield increased the SG during selection. Thus, newer hybrids [140] and newer populations [141] are more SG than the old ones; that is, the increased level of delayed senescence of newer hybrids contribute to their higher productivity (Figure 4) [15,142]. Moreover, SG maize genotypes had a high tolerance to abiotic stresses (such as drought and high population density) compared to non-GS genotypes. 

Cerrudo [139], laid out the basics to utilize high throughput phenotyping and facilitated the identification of climate-adapted germplasm. To identify and select germplasm with high grain yield under drought, heat, and combined drought-heat stresses, starting at anthesis, they used an airplane mounted multispectral camera to estimate the area under the curve (AUC) for vegetation indices to measure and compute secondary traits. Among the secondary traits, NDVI was found to be the best secondary trait to breed for high grain yield and extended SG under drought, heat, and combined drought-heat stresses [139]. Furthermore [139] found that the prediction accuracy of the secondary traits like NDVI was better than the prediction accuracy of grain yield under stress and non-stress conditions. NDVI is a highly heritable trait with moderate and consistent correlation with grain yield under well-watered conditions [138,143]. 

Most of SG genes have been identified and functionally characterized in maize and rice (Table 1), work in sorghum and wheat is still behind, although in sorghum the trait extensively studied and sorghum genome was sequenced. Genes were identified, but the SG related function still un-known.

## 6. Stay-Green in Barley

### 6.1. QTLs for the Stay-Green Trait in Barley

Unlike other cereal crops, SG research in barley is very limited. Emeberi [54] in multiple barley populations identified nine QTLs related to SG. Of these nine QTLs, only that on the short arm of barley chromosome 5H showed a consistency under different environments in all populations; however, its expression possessed high GxE interaction. On the other hand, the presence of only one consistent QTL suggests that the leaf senescence/Chl loss during the maturity stage is controlled by simple genetic factors [54]. Sallam [144] found that QTLs for leaf rolling and leaf chlorophyll content on chromosome two, four, and five are syntenic between barley and wheat. Obsa [145] evaluated three interconnected doubled haploid populations in drought-prone environments and detected 18 QTLs for drought adaptation. Among these 18 QTLs, four and two QTLs were detected for SG related traits NDVI and SPAD, respectively. Fox [146] evaluated the SG expression under terminal heat and drought stresses in 100 barley lines from a ND24260 × Flagship doubled haploid population. They detected ten SG QTLs on chromosomes 3H, 4H, 5H, 6H, and 7H. Out of these ten QTLs, six QTLs were associated with terminal heat-stress and four with terminal drought stress. These QTLs did not co-localized with previously reported barley stress-response QTL and therefore considered as novel QTLs. However, the two heat-stress QTLs mapped to bPb-5529 on chromosome 5H, are near to QTLs reported for root/shoot ratio and root length [146]. After field validation these identified QTLs could be good candidates for MAS targeting improvement of barley abiotic stress tolerance. 

### 6.2. The Physiology of Stay-Green in Barley

Although drought-tolerant barley has been reported by Gonzalez [147] very few reports discussed the SG trait. SG reported frequently for leaf greenness while other organs contribution was detected. CO_2_ estimates indicate that the spikes’ contribution to grain yield can reach up to 70% depending on the conditions in wheat and barley grown under stress [148]. Vaezi1 [149], found that among 11 barley genotypes evaluated under drought stress, the highest yielding genotype possess SG characteristics. Therefore, they suggested that potential grain yield can be improved by increasing plant photosynthetic capacity and assimilates production during the later phase of grain filling. Seiler [150] studied a number of barley lines showing senescence or SG phenotype and demonstrated the superior yield performance of the SG lines under drought conditions. They found that the reason of the difference between the SG and senescing lines in their assimilation capacity under drought stress is that the ABA synthesis levels in senescing lines are greater than that of SG lines under short and long-term drought stress. Based on this finding they suggested that a greater ABA flux metabolism in the senescing lines negatively affected assimilation and water use efficiency [150]. Shirdelmoghanloo [151] studied 157 barley genotypes under heat stress in two environments comprising three sowing dates and detected genetic variation for grain growth components, grain plumpness, and SG traits. Their results showed a significant positive correlation between the SG and the grain filling duration which suggests the role of the SG in stabilization of the grain filling duration in barley under heat stress. Moreover, they demonstrated the possibility of developing heat-tolerant barley genotypes through appropriate focus on grain filling rate and SG traits in breeding programs [151].

## 7. Stay-Green and Grain or End-Use Quality

Although, grain quality or end-use quality in cereals is an important aspect and very hot topic in the research community, knowledge on the effects of “SG” expression on grain or end-use quality under normal or stress conditions is extremely limited. On the other hand, as it has been reported that SG trait is capable of protecting the cereal yields under stress conditions through organization and stabilization of grain development, it is possible to speculate that it might have a positive impact on the grain or end-use quality. In sorghum, SG plants showed increased resistance to pest and disease invasion, better quality forages for animals, high chl content, and extended pigment source for food industry, as well as the attractive ornamental period [44]. In study of 50 winter wheat genotypes, a negative correlation between the onset of leaf senescence and grain yield (*r*^2^ = 0.81) and a positive correlation with grain protein content (*r*^2^ = 0.48) was observed [89]. It is concluded that the SG phenotypes were characterized by higher N uptake during grain filling and longer maintenance of greenness. In addition, the use of photosynthetic glucose for the synthesis of amino acids rather than for starch decreased wheat yield and increased grain protein content [89]. NDVI measurements using a drone to estimate the SG phenology have recently been demonstrated to improve wheat grain quality and yield predictions [152] In rice, it has been speculated that delaying the senescence at the terminal stage of maturity may lead to increased yield and improved grain quality [95] In barley, Gous [153] examined the effect of SG expression on starch biosynthesis in grains of Flagship (a cultivar without “SG”-like characteristics) and ND24260 (SG”-like cultivar) under mild and severe drought stress conditions at anthesis. In this study Flagship possessed higher grain amylose and long amylopectin branches under the mild drought stress, suggesting that drought stress affects starch biosynthesis in grain, probably because of early termination of grain filling. In contrast, ND24260 did not possess any changes in starch molecular structure under the different drought levels. As long as changes in starch molecular structure can affect starch properties, such as enzymatic degradation rates, and hence its nutritional value, the ND24260 has a greater potential to maintain starch biosynthesis and hence better grain quality under drought conditions. These results make the “SG”-like traits potentially useful to ensure food quality and quantity [153].

## 8. Conclusions and Future Prospective

This review discussed the recent progress made in the research of the SG as an important trait to combat abiotic stresses in the major cereals. The review discussed the identification of SG and SG-related traits QTLs and the SG genes. The five cereal crops differ in their chromosome numbers and genome sizes; however, they have been diverged from common ancestor 60 million years ago [165] and there is a degree of synteny between their genomes [166] and [22]. Considering the synteny between cereals genomes, we suggest that comparative mapping approaches using the huge genomic information became available recently would elucidate inheritance, physiology, and expression of SG, and would generate massive genetic information regarding the SG including identification of the genes leading to full understanding of the SG mechanism. Interestingly this can be done without extensive phenotyping.

Despite the knowledge of genomic regions conferring the SG trait, it is surprising that knowledge about the physiological mechanisms of the SG is still relatively limited. Early explanations focused mainly on the role of SG in the maintenance of photosynthetic activity. However, the SG trait has been suggested to be involved (regulates) in (1) the plant N/C balance and in particular to increase the capacity of C capture and N mobilization during the post-anthesis period; (2) increased water availability during the post-anthesis period. However, the improvements in water uptake and water use efficiency because of SG phenotype are still unexplained, which could be accounted for either a deeper soil extraction because of improved root architecture or water-saving traits operating at early stages. This could be a useful future study to elucidate the water relations in the SG plants for efficient cereal breeding under drought. The strong association between SG QTLs in major cereal crops and other useful agronomic traits such as grain yield improvement, sunlight interception, and conversion and biomass allocation, especially under drought and heat stress conditions, would also provide opportunities to use both phenotypic and molecular markers of SG trait to accelerate the breeding of new cereal varieties. 

The studies reported here support the use of leaf and canopy photosynthesis, as a target trait to breed high-yielding cultivars. High yielding genotypes adopt different strategies to achieve high production, and this can explain the complexity of grain yield formation under favorite and stress conditions. This review provides evidence that there is a need to phenotype photosynthetic capacity-related traits, leaf anatomy, canopy development at vegetative, pre, and post-anthesis stages. As the SG mechanisms become more evident and as DNA-sequencing offers intensive genome coverage, the possibility is that the future of manipulating the SG trait will be about manipulating its physiological components. Thus, this review has contributed to increasing knowledge on the understanding of the physiological mechanisms associated with SG trait and photosynthetic efficiency in cereals as a prospective approach for high yield under stress conditions. Maybe the key to breaking the plateau of productivity associated with adaptation to stress conditions particularly heat and drought stresses. More exploration needs to be done extensively in breeding programs to benefit from this trait like gene identifications, grain quality, and deep physiological analysis, including metabolomic, transcriptomic and ionomic studies. This trait in combination with other useful traits may provide the solution against the major environmental problems (heat and drought). Information about QTLs for SG trait in major cereal crops would also provide opportunities to use this trait in breeding programs.

At last, taking into account the knowledge accumulated about the SG in the five cereal crops and the synteny between them we can describe the SG as plant mechanism manage the canopy size, water uptake and utilization, nitrogen and carbon dynamics, leaf senescence, photosynthesis capacity, and finally assimilates partitioning. All these comprise very complicated and interacted biochemical processes through hormonal balance and other bath ways. Based on this, it is obvious that SG is a very complicated trait and to be more precise SG should be nominated as a system and not just a trait.

## Figures and Tables

**Table 1:** List of stay-green-related genes and their functional characterization in the four major cereal crops (Rice, Wheat, Maize, and Sorghum).

Genes	Function	Cereals				References
		Rice	Wheat	Maize	Sorghum	
Stay-Green Rice like (*SGRL*)	Affect Chlorophyll (Chl) degradation during natural and dark-induced leaf senescence	√	-	-	0	[116,154]
Glucuronic acid substitution of xylan1 (*GUX1*)	Required for substitution of the xylan backbone with 4-O-methylglucuronic acid [Me]GlcA	-	0	√	0	[125,155,156,157,158]
β-Glucosidase (*BGLU42*)	The exact role remains to be determined	√	-	√	0	[125]
Trehalose-6-phospate synthase13(*trps13*)	Sugar-mediated signaling in stay-green	-	-	√	0	[125]
Monosaccharide transporter (*MST4*)	Associated with senescence and nitrogen use efficiency, transport of Sucrose out of leaf cells by sugar transporters to alternative sinks	√	-	√	-	[125,159]
Sugar will Eventually be exported transporters (*SWEET*)	Transport of Sucrose out of leaf cells by sugar transporters to alternative sinks	√	-	√	-	[125,160]
Cell wall invertase (*incw4*)	Sinks hydrolyzation	-	-	√	0	[125,161]
Mitochondrial pentatricopeptide repeat protein (*dek10*)	Activation sugar-sinks	-	-	√	-	[125,162,163]
*Sb09g004170* and *Sb09g022580*	DRGs (Associated with *Stg1* QTL)	-	-	-	√	[164]
NAC-transcription factor 9(*nactf9*)	Appears to be one of the master regulators of stay-green, acting in conjunction with ZmIRX15-L, ZmGUX1, mlg3	-	-	√	-	[125]

√, -, 0: Identified and characterized, not identified yet, identified, however, their contribution to the stay-green trait not yet known.

## Abbreviations

SG	Stay-green
Chl	Chlorophyll
NDVI	Normalized difference vegetation index
CTD	Canopy temperature depression
MAS	Marker-assisted selection
